# Role of the C‐terminus of SUR in the differential regulation of β‐cell and cardiac K_ATP_ channels by MgADP and metabolism

**DOI:** 10.1113/JP276708

**Published:** 2018-10-14

**Authors:** Natascia Vedovato, Olof Rorsman, Konstantin Hennis, Frances M. Ashcroft, Peter Proks

**Affiliations:** ^1^ Henry Wellcome Centre for Gene Function Department of Physiology Anatomy and Genetics University of Oxford Parks Road Oxford OX1 3PT UK

**Keywords:** SUR1, SUR2A, KATP channel, channel regulation

## Abstract

**Key points:**

- β‐Cell K_ATP_ channels are partially open in the absence of metabolic substrates, whereas cardiac K_ATP_ channels are closed.
- Using cloned channels heterologously expressed in *Xenopus* oocytes we measured the effect of MgADP on the MgATP concentration–inhibition curve immediately after patch excision.
- MgADP caused a far more striking reduction in ATP inhibition of Kir6.2/SUR1 channels than Kir6.2/SUR2A channels; this effect declined rapidly after patch excision.
- Exchanging the final 42 amino acids of SUR was sufficient to switch the Mg‐nucleotide regulation of Kir6.2/SUR1 and Kir6.2/SUR2A channels, and partially switch their sensitivity to metabolic inhibition.
- Deletion of the C‐terminal 42 residues of SUR abolished MgADP activation of both Kir6.2/SUR1 and Kir6.2/SUR2A channels.
- We conclude that the different metabolic sensitivity of Kir6.2/SUR1 and Kir6.2/SUR2A channels is at least partially due to their different regulation by Mg‐nucleotides, which is determined by the final 42 amino acids.

**Abstract:**

ATP‐sensitive potassium (K_ATP_) channels couple the metabolic state of a cell to its electrical activity and play important physiological roles in many tissues. In contrast to β‐cell (Kir6.2/SUR1) channels, which open when extracellular glucose levels fall, cardiac (Kir6.2/SUR2A) channels remain closed. This is due to differences in the SUR subunit rather than cell metabolism. As ATP inhibition and MgADP activation are similar for both types of channels, we investigated channel inhibition by MgATP in the presence of 100 μm MgADP immediately after patch excision [when the channel open probability (*P*_O_) is near maximal]. The results were strikingly different: 100 μm MgADP substantially reduced MgATP inhibition of Kir6.2/SUR1, but had no effect on MgATP inhibition of Kir6.2/SUR2A. Exchanging the final 42 residues of SUR2A with that of SUR1 switched the channel phenotype (and vice versa), and deleting this region abolished Mg‐nucleotide activation. This suggests the C‐terminal 42 residues are important for the ability of MgADP to influence ATP inhibition at Kir6.2. This region was also necessary, but not sufficient, for activation of the K_ATP_ channel in intact cells by metabolic inhibition (azide). We conclude that the ability of MgADP to impair ATP inhibition at Kir6.2 accounts, in part, for the differential metabolic sensitivities of β‐cell and cardiac K_ATP_ channels.

## Key points

- β‐Cell K_ATP_ channels are partially open in the absence of metabolic substrates, whereas cardiac K_ATP_ channels are closed.
- Using cloned channels heterologously expressed in *Xenopus* oocytes we measured the effect of MgADP on the MgATP concentration–inhibition curve immediately after patch excision.
- MgADP caused a far more striking reduction in ATP inhibition of Kir6.2/SUR1 channels than Kir6.2/SUR2A channels; this effect declined rapidly after patch excision.
- Exchanging the final 42 amino acids of SUR was sufficient to switch the Mg‐nucleotide regulation of Kir6.2/SUR1 and Kir6.2/SUR2A channels, and partially switch their sensitivity to metabolic inhibition.
- Deletion of the C‐terminal 42 residues of SUR abolished MgADP activation of both Kir6.2/SUR1 and Kir6.2/SUR2A channels.
- We conclude that the different metabolic sensitivity of Kir6.2/SUR1 and Kir6.2/SUR2A channels is at least partially due to their different regulation by Mg‐nucleotides, which is determined by the final 42 amino acids.

## Introduction

ATP‐sensitive potassium (K_ATP_) channels couple the metabolic state of a cell to its electrical activity. They consist of pore‐forming Kir6.x subunits and regulatory sulphonylurea receptor (SURx) subunits, both of which participate in metabolic regulation of channel activity (Rorsman & Ashcroft, 2018). Kir6.2/SUR1 channels link blood glucose levels to insulin secretion from pancreatic β‐cells and regulate transmitter release in neurones; Kir6.2/SUR2A channels are involved in the response to cardiac stress; and Kir6.1/SUR2B channels regulate vascular smooth muscle tone (Nichols & Lederer, 1991; Daut *et al*. 1994; Quayle *et al*. 1997; Ashcroft & Gribble, 1999; Seino *et al*. 2000).

Metabolic regulation of channel activity is mediated by changes in the cytosolic concentration of adenine nucleotides, which interact with nucleotide‐binding sites on both subunits. Binding of ATP (or ADP) to Kir6.x produces channel inhibition, whereas interaction of MgADP (or MgATP) with the nucleotide‐binding domains (NBDs) of SUR stimulates channel activity (Nichols *et al*. 1996; Gribble *et al*. 1997; Shyng *et al*. 1997; Tucker *et al*. 1997). The NBDs come together in a sandwich dimer fashion to form two nucleotide binding sites (NBS) at the interface (Lee *et al*. 2017). When examined in excised patches, little difference is observed in the sensitivity of Kir6.2/SUR1 and Kir6.2/SUR2A channels to inhibition by MgATP (Shyng *et al*. 1997; Gribble *et al*. 1998; Abraham *et al*. 2002), or in channel activation by MgADP (Hopkins *et al*. 1992; Nichols *et al*. 1996; Gribble *et al*. 1997; Dupuis *et al*. 2008; Proks *et al*. 2010, 2013). Nevertheless, these channels exhibit very different sensitivities to metabolism in intact cells. For example, in β‐cells (Kir6.2/SUR1) and microvascular coronary endothelial cells (Kir6.1/SUR2B) K_ATP_ channels open when extracellular glucose levels fall (Ashcroft *et al*. 1984; Langheinrich & Daut, 1997), whereas cardiac channels (Kir6.2/SUR2A) remain closed in glucose‐free solutions and only open in response to severe metabolic inhibition (Nichols & Lederer, 1991; Flagg *et al*. 2010). Even when expressed in the same cell type, Kir6.2/SUR1, but not Kir6.2/SUR2A, channels are activated by metabolic poisoning (Dabrowski *et al*. 2001; Clark *et al*. 2010; Li *et al*. 2016).

The cause of these differences in metabolic sensitivity remains unclear. However, it is of important functional and clinical significance. It explains, for example, why patients or mice with gain‐of‐function mutations in Kir6.2 develop neonatal diabetes but have no obvious impairment in cardiac or skeletal muscle function (Clark *et al*. 2010, 2012). In this paper, we therefore examine how SUR1 and SUR2A confer different metabolic sensitivities upon Kir6.2. We find striking differences in MgATP inhibition of Kir6.2/SUR1 and Kir6.2/SUR2A channels in the presence of MgADP. We further show that these differences in nucleotide handling can be accounted for by differences in the C‐terminus of SUR. However, they only partially account for the different metabolic sensitivities of these channels.

## Materials and Methods

### Ethical approval

Experiments on *Xenopus leavis* oocytes were conducted in accordance with the policies and regulations set out in ASPA Schedule 1 in the UK and *The Journal of Physiology*’s guidelines on animal ethics. Animals were purchased from the University of Portsmouth (Portsmouth, UK) and housed in the University of Oxford's animal facility, where they were fed twice a day and kept in filtered water. They were killed with an overdose of ethyl‐m‐aminobenzoate methanesulphonate (MP Biomedicals, LLC, CA, USA). Once anaesthesia was complete (as assessed by loss of reflexes), animals were killed by brain stem disruption. Oocytes were then removed under sterile conditions.

### Molecular biology

Mouse Kir6.2 (GenBank NM010602), rat SUR1 (GenBank L40624), rat SUR2A (GenBank D83598) and rat SUR2B (GenBank Q9JJ67) were used in this study. Site‐directed mutagenesis and preparation of mRNA were performed as described previously (Proks *et al*. 2005). Oocytes were injected with 0.8 ng wild‐type or mutant Kir6.2 mRNA and ∼4 ng SUR mRNA, and currents were recorded 1–4 days after injection.

### Surface expression

Surface expression assays were performed 2–3 days after injection of Kir6.2/SURx with or without a haemagglutinin (HA) label in the extracellular loop of Kir6.2, as described previously (Zerangue *et al*. 1999). Briefly, oocytes were incubated for 30 min in ND96 with 1% bovine serum albumin (BSA) at 4°C to block non‐specific antibody binding, labelled with 1 μg/ml rat monoclonal anti‐HA antibody (3F10, Boehringer Mannheim, Mannheim, Germany, in ND96−1% BSA for 40 min at 4°C), washed at 4°C for 1 h, and incubated with 2 μg/ml horseradish peroxidase‐coupled secondary antibody in ND96−1% BSA for 30 min at 4°C. Cells were extensively washed, first with ND96−1% BSA and then in ND96 without BSA at room temperature (both washes for 40 min). Each individual oocyte was placed in 50 μl Power Signal Elisa (Pierce, Rockford, IL, USA) at room temperature and chemiluminescence was quantified in a Glomax 20/20 luminometer.

### Two‐electrode voltage‐clamp recordings

Whole‐cell currents were recorded from intact oocytes in response to voltage steps of ±20 mV from a holding potential of −10 mV, filtered at 0.5 kHz and digitised at 4 kHz. Oocytes were continuously perfused at room temperature with solution containing (mm): 90 KCl, 1 MgCl_2_, 1.8 CaCl_2_ and 5 Hepes, pH 7.4 with KOH. Metabolic inhibition was induced with 3 mm sodium azide. Currents were activated by the K‐channel openers diazoxide (340 μm; Kir6.2/SUR1 channels) or pinacidil (100 μm; Kir6.2/SUR2A), and inhibited by the sulphonylurea tolbutamide (Kir6.2/SUR1, 0.5 mm) or glibenclamide (Kir6.2/SUR2A, 50 μm).

### Patch‐clamp recordings and data analysis

Currents were recorded as previously described (Proks *et al*. 2005) from either giant inside‐out or cell‐attached patches pulled from *Xenopus* oocytes at −60 mV, filtered at 5 kHz and digitised at 20 kHz. The pipette solution contained (mm): 140 KCl, 1.2 MgCl_2_, 2.6 CaCl_2_, 10 Hepes (pH 7.4 with KOH). The intracellular (bath) solution contained (mm): 107 KCl, 1 CaCl_2_, 2 MgCl_2_, 10 EGTA, 10 Hepes (pH 7.2 with KOH to a total of K^+^ concentration of ∼140 mm) and MgATP or MgADP as indicated. The Mg‐free intracellular solution contained (mm): 107 KCl, 1 K_2_SO_4_, 10 EGTA, 10 Hepes (pH 7.2 with KOH; total [K^+^] ∼140 mm) and K_2_ATP or K_2_ADP as indicated. Experiments were conducted at room temperature.

K_ATP_ channels in excised patches, whether from pancreatic β‐cells, mammalian cell lines or *Xenopus* oocytes, undergo both fast and slow rundown (reviewed by Proks *et al*. 2016). The nucleotide sensitivity of K_ATP_ channels was assessed either as close to patch excision as possible (‘instantaneous’) or after fast rundown was complete (‘rundown’). To measure the instantaneous ATP sensitivity, a single ATP concentration was applied per patch, in either the presence or the absence of 100 μm MgADP. In all cases, the current in the test solution was expressed as a fraction of the mean of that in control solution before and after ATP application.

The relationship between nucleotide concentration and K_ATP_ current inhibition was fitted with:

(1) $$\frac{I_{x}}{I_{o}}=\frac{1}{1+{\left(\frac{\left[X\right]}{IC_{50}}\right)}^{h}}$$

where *I_X_* is the steady‐state K_ATP_ current in the presence of the test nucleotide concentration [*X*], *I*_0_ is the current in nucleotide‐free solution obtained by averaging the current before and after application, *IC*_50_ is the nucleotide concentration at which the inhibition is half maximal and *h* is the Hill coefficient.

The single‐channel open probability (*P*_O_) in cell‐attached patches containing a small number of channels (*N *< 8) was estimated from *NP*_O_, as described previously (Proks *et al*. 2005). For cell‐attached patches containing larger number of channels, *P*_O_ was estimated as:

(2) $$P_{o}=\frac{I_{MEAN}}{Ni}$$

where *I*_MEAN_ is the mean K_ATP_ current in the cell‐attached configuration, *N* is the number of active channels in the patch and *i* is the single‐channel current (*i *= 4 pA at−60 mV). Following cell‐attached recordings, the patch was excised and the number of active channels (*N*) was estimated using noise analysis from ∼1 s data stretches obtained after the K_ATP_ current had reached its maximum (Proks *et al*. 2010). The value of *N* determined by noise analysis varied between 8 and 750. There was no obvious difference between *P*_O_ values determined by *NP*_O_ analysis and noise analysis and there was no obvious relationship between *P*_O_ and *N*. The latter indicates that it is unlikely that the value of *P*_O_ is distorted when calculated by noise analysis.

### Statistics

All values are given as mean ± SEM. Statistical significance was determined using Student's *t*‐test.

## Results

### Instant *vs*. rundown MgATP sensitivity

Concentration–response relationships for nucleotide regulation of K_ATP_ channel activity have normally been measured some time after patch excision. As channel activity usually runs down in excised patches, this inevitably means these studies have reported the nucleotide sensitivity of rundown or partially rundown channels. Both rundown and the decline of activation by magnesium nucleotides (DAMN) have been proposed to affect K_ATP_ channel ATP sensitivity (Ribalet *et al*. 2000; Proks *et al*. 2010). Thus it is possible that differences in rundown, or DAMN, between Kir6.2/SUR1 and Kir6.2/SUR2A channels may explain the apparent discrepancy between nucleotide sensitivity in the intact cell and excised patch.

**Figure 1:**
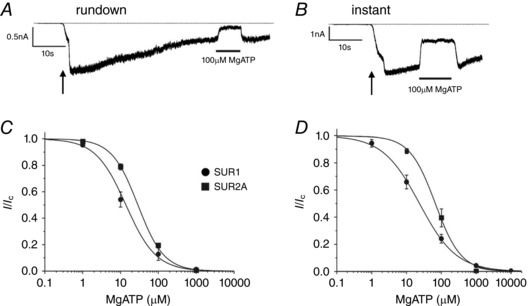
MgATP sensitivity of Kir6.2/SUR1 and Kir6.2/SUR2A channels Representative traces showing time points at which rundown (*A*) and instantaneous (*B*) ATP sensitivities were measured for Kir6.2/SUR1. The arrow indicates patch excision. In the case of the instantaneous concentration–response relationship only a single ATP concentration was tested per patch, whereas a full concentration–response relationship was performed in each patch following rundown. *C* and *D*, rundown (*C*) and instantaneous (*D*) concentration–response relationships for MgATP inhibition of Kir6.2/SUR1 (circles) or Kir6.2/SUR2A channels (squares). Current is measured in excised patches and expressed relative to that in the absence of nucleotide. The continuous lines are the best fit of eqn (1) to the mean data. *C*: ●, *IC*_50_ = 13.9 μm, *h* = 1.1 (*n* = 8); ■, *IC*_50_ = 28.7 μm, *h* = 1.3 (*n* = 8). *D*: ●, *IC*_50_ = 23.6 μm, *h* = 0.82 (*n* = 5); ■, *IC*_50_ = 66.8 μm, *h* = 1.2 (*n* = 5).

We therefore compared the ability of MgATP to inhibit Kir6.2/SUR1 and Kir6.2/SUR2 channels immediately after patch excision (see Methods) and after rundown (Table 1). The *IC*_50_ for rundown channels was similar to that reported previously (Nichols *et al*. 1991; Drain *et al*. 1998; Gribble *et al*. 1998; Tarasov *et al*. 2006; Clark *et al*. 2010). The *IC*_50_ for instantaneous ATP inhibition (Fig. 1 *B*) was approximately twice that measured following rundown (Fig. 1 *A*), for both types of channel. Under both conditions, cardiac K_ATP_ channels were slightly less inhibited by MgATP than β‐cell K_ATP_ channels.

**Table 1:** Nucleotide inhibition of K_ATP_ channels with different SUR subunits

			*IC*_50_ (μm)		
	Mg^2+^	MgADP (μm)	SUR1	SUR2A	SUR2B
Instantaneous	+	0	24 ± 2	67 ± 6	100 ± 3
Instantaneous	+	100	504 ± 26	65 ± 3	355 ± 2
Rundown	−	0	6.5 ± 0.5	7.7 ± 0.8	n.d.
Rundown	+	0	14 ± 2	29 ± 4	117*
Rundown	+	100	120 ± 9	59 ± 6	n.d.

Fitting parameters for ATP concentration–inhibition relationships. ^*^Data from Reimann *et al*. (2000). n.d., not done. SUR was coexpressed with Kir6.2.

### ATP in the presence of MgADP

**Figure 2:**
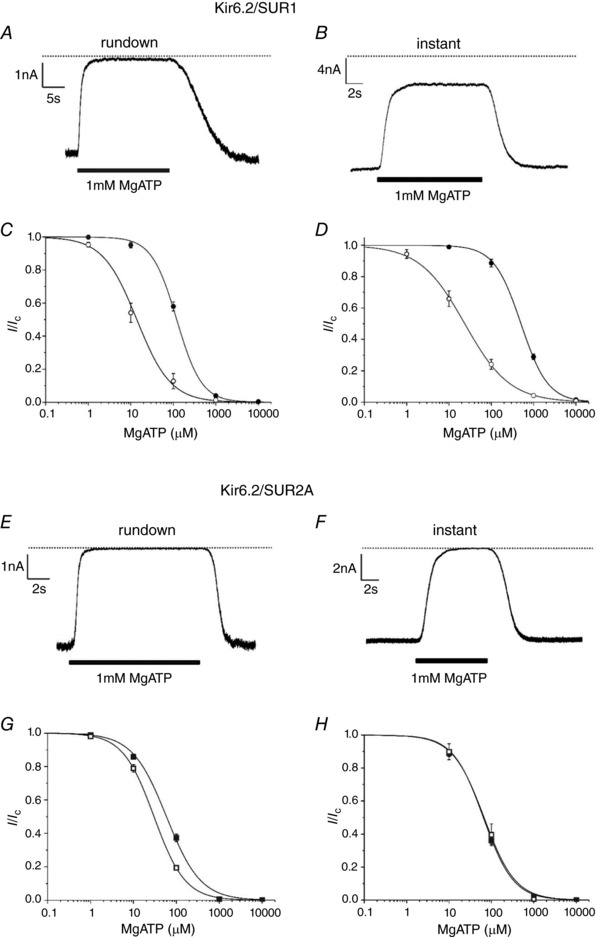
Effects of MgADP on MgATP inhibition of Kir6.2/SUR1 and Kir6.2/SUR2A channels *A* and *B*, representative traces of Kir6.2/SUR1 currents recorded in the continuous presence of 100μM MgADP and (as indicated) 1mM MgATP following rundown (*A*) or immediately after patch excision (*B*). Rundown (*C*) and instantaneous (*D*) concentration–response relationships for MgATP inhibition of Kir6.2/SUR1 channels in the absence (open symbols) and presence (filled symbols) of 100 μm MgADP. *C*: ○, *IC*_50_ = 14 μm, *h* = 1.1 (*n* = 8); ●, *IC*_50_ = 120 μm, *h* = 1.5 (*n* = 6). *D*: ○, *IC*_50_ = 24 μm, *h* = 0.83 (*n* = 6); ●, *IC*_50_ = 504 μm, *h* = 1.4 (*n* = 6). *E* and *F*, representative traces of Kir6.2/SUR2A currents recorded in the continuous presence of 100 μM MgADP and (as indicated) 1 mM MgATP following rundown (*E*) or immediately after patch excision (*F*). Rundown (*G*) and instantaneous (*H*) concentration–response relationships for MgATP inhibition of Kir6.2/SUR2A channels in the absence (open symbols) and presence (filled symbols) of 100 μm MgADP. *G*: □, *IC*_50_ =  29€μm, *h* = 1.3 (*n* = 6); ■, *IC*_50_ = 59 μm, *h* = 1.1 (*n* = 6). *H*: □, *IC*_50_ = 67 μm, *h* = 1.2 (*n* = 6); ■, *IC*_50_ = 64 μm, *h* = 1.2 (*n* = 6). Currents are expressed relative to that in the absence of MgATP. The continuous lines are the best fit of eqn (1) to the mean data.

In the intact cell, both MgATP and MgADP are present simultaneously so that K_ATP_ channel activity will be determined by the balance between nucleotide inhibition at Kir6.2 and nucleotide activation at SUR. We therefore next examined the effect of MgADP on both the rundown and the instantaneous MgATP concentration–response curves. We used 100 μm MgADP, which is close to that found in oocytes treated with sodium azide (97 μm; Li *et al*. 2016). MgADP reduced the channel sensitivity to MgATP to an extent that depended on both the type of SUR subunit and the time after patch excision at which measurements were made (Fig. 2, Table 1).

**Figure 3:**
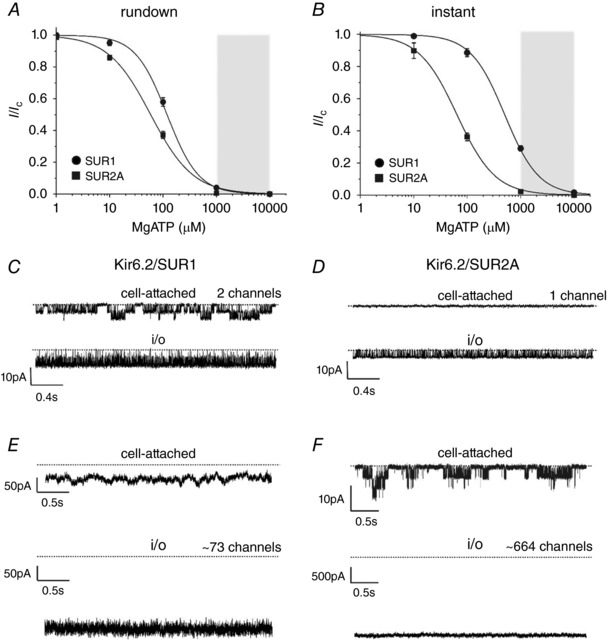
Comparison of nucleotide sensitivity in inside‐out and cell‐attached patches *A* and *B*, MgATP inhibition of Kir6.2/SUR1 (●) and Kir6.2/SUR2A (■) channels in the presence of 100 μm MgADP measured after rundown (*A*) or immediately after patch excision (*B*). Data as in Fig. 2. The grey box indicates the physiological range of MgATP concentrations (1–10 mm). *C*–*F*, representative K_ATP_ currents recorded at −60 mV in a cell‐attached (above) and subsequently inside‐out (below) patch from *Xenopus* oocytes expressing Kir6.2/SUR1 (*C*,*E*) or Kir6.2/SUR2A (*D*,*F*). Oocytes were preincubated in 3 mm azide for 30 min prior to the recording. The number of channels in *E* and *F* was estimated by noise analysis (see Methods). The dotted line represents the zero current level.

MgADP (100 μm) produced an increase in the *IC*_50_ for ATP inhibition measured after rundown that was much greater for Kir6.2/SUR1 than for Kir6.2/SUR2A (compare Fig. 2 *C*,*G*). The difference was even more dramatic for the instantaneous ATP concentration–response relationship. Whereas there was a very large reduction in ATP sensitivity in the presence of MgADP for Kir6.2/SUR1 channels (Fig. 2 *D*), no difference was observed for Kir6.2/SUR2A channels (Fig. 2 *H*). Direct comparison of the instantaneous ATP concentration–response relationships in the presence of MgADP for Kir6.2/SUR1 *vs*. Kir6.2/SUR2A channels (Fig. 3 *B*) clearly revealed that Kir6.2/SUR2A channels are almost completely closed at physiological MgATP concentrations (>1 mm), whereas ∼30% of the Kir6.2/SUR1 current remains uninhibited. This result was completely missed when comparing the rundown ATP concentration–response relationships (Fig. 3 *A*).

Cell‐attached recordings in the presence of 3 mm sodium azide revealed significant on‐cell channel activity in oocytes expressing Kir6.2/SUR1 (Fig. 3 *C*,*E*). The mean single‐channel open probability (*P*_O_) was 0.26 ± 0.03 (*n *= 30). There was no obvious correlation between the estimated *P*_O_ in cell‐attached recordings and the number of active channels in the patch (*N*). Given that the maximal open probability of Kir6.2/SUR1 channels was 0.86 (as estimated by noise analysis of the peak current after excision; Table 2), this predicts that the channels remained ∼30% uninhibited (in the presence of azide), which is close to that obtained in excised patches exposed to 1 mm MgATP and 100 μm MgADP (29%; Fig. 3 *A*).

**Table 2:** Single‐channel open probability (*P*_O_) of K_ATP_ channels in inside‐out patches

	*P*_O_	Kir6.2/SUR1	Kir6.2/SUR2A	Kir6.2/SUR1Δ42	Kir6.2/SUR2Δ42
Instantaneous	Control	0.86 ± 0.01 (*n* = 6)	0.86 ± 0.01 (*n* = 6)	0.62 ± 0.06 (*n* = 6)	0.66 ± 0.07 (*n* = 6)
Instantaneous	100μm MgADP	0.84 ± 0.01 (*n* = 6)	0.84 ± 0.01 (*n* = 6)	n.d.	n.d.
Rundown	Control	0.41 ± 0.02 (*n* = 10)	0.71 ± 0.02 (*n* = 10)	n.d.	n.d.

*P*_O_ was estimated using noise analysis. n.d., not done.

In contrast to Kir6.2/SUR1, most (62%) cell‐attached patches on oocytes expressing Kir6.2/SUR2A showed no detectable channel activity, although some channel activity was observed after patch excision (Fig. 3 *D*). In those patches where single Kir6.2/SUR2A channel currents were detected, very large currents were observed after patch excision (Fig. 3 *F*). This suggests the existence of a small fraction of Kir6.2/SUR2A channels with reduced sensitivity to nucleotide inhibition.

Taken together, these data suggest that the difference in the metabolic sensitivity of Kir6.2/SUR1 and Kir6.2/SUR2A channels may be related to the differences in nucleotide handling.

### What causes the difference in nucleotide handling?

Matsuoka *et al*. (2000) showed that replacement of the final 42 residues of SUR2A with those of SUR1 enhanced the ability of MgADP to activate channels inhibited by 1 mm MgATP. Conversely, in the presence of 1 mm MgATP, MgADP activation was dramatically suppressed when the C‐terminal 42 residues of SUR1 were replaced with those of SUR2A (Matsuoka *et al*. 2000).

**Figure 4:**
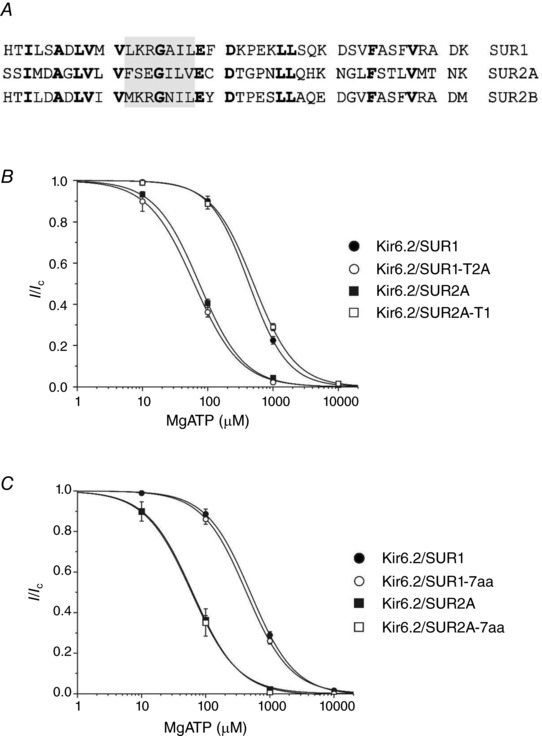
The C‐terminus modulates nucleotide interactions with the K_ATP_ channels (data from instantaneous measurements in inside‐out patches) *A*, sequence alignment of the C‐termini of rat SUR1, rat SUR2A and rat SUR2B. The grey box indicates the 7 amino acids previously identified by Matsushita *et al*. (2002). *B*, concentration–response relationships for MgATP inhibition of Kir6.2/SUR1 (●), Kir6.2/SUR2A (■), Kir6.2/SUR1‐T2A (○) and Kir6.2/SUR2A‐T1 (□) channels measured in the presence of 100 μm MgADP immediately after patch excision. ●, *IC*_50_ = 504 μm, *h* = 1.4 (*n* = 6); ■, *IC*_50_ = 58.8 μm, *h* = 1.1 (*n* = 6); ○, *IC*_50_ = 430 μm, *h* = 1.5 (*n* = 6); □, *IC*_50_ = 74.4 μm, *h* = 1.3 (*n* = 6). *C*, concentration–response relationships for MgATP inhibition of Kir6.2/SUR1 (●), Kir6.2/SUR2A (■), Kir6.2/SUR1‐7aa (○) and Kir6.2/SUR2A‐7aa (□) channels measured in the presence of 100 μm MgADP immediately after patch excision. ●, *IC*_50_ = 504 μm, *h* = 1.4 (*n* = 6); ■, *IC*_50_ = 58.8 μm, *h* = 1.1 (*n* = 6); ○, *IC*_50_ = 430 μm, *h* = 1.3 (*n* = 6); □, *IC*_50_ = 63 μm, *h* = 1.2 (*n* = 6).

However, Matsuoka *et al*. (2000) only investigated the effect of MgADP at a single ATP concentration. This paper also did not assess the instantaneous response to nucleotides, which may more closely resemble that found in the intact cell. We therefore examined the effect of exchanging the last 42 amino acids (the ‘tail’; Fig. 4 *A*) of SUR1 and SUR2A on the instantaneous MgATP concentration–response relationship in the presence of MgADP. As shown in Fig. 4 *B*, the instantaneous concentration–response relationship was identical for SUR1 with the tail of SUR2A (SUR1‐T2A) and for SUR2A. Conversely the tail of SUR1 endowed SUR2A (SUR2A‐T1) with the properties of SUR1. This confirmed that the final 42 amino acids play a key role in determining the difference in nucleotide activation of SUR1 and SUR2A.

Unlike Kir6.2/SUR2A, Kir6.2/SUR2B channels can be activated by metabolic inhibition with sodium azide (Li *et al*. 2016), despite the fact that SUR2A and SUR2B differ only in their last 42 amino acids (Fig. 4 *A*). Furthermore, 100 μm MgADP caused a marked reduction in the instantaneous ATP sensitivity of Kir6.2/SUR2B channels (Table 1), as it does for Kir6.2/SUR1. The difference in MgADP activation of Kir6.2/SUR2A and Kir6.2/SUR2B channels in the presence of MgATP is reported to be due to 7 amino acids (residues 1516–1522, grey box in Fig. 4 *A*, 7aa) in the C‐terminus of SUR2 (Matsushita *et al*. 2002). However, exchanging these amino acids between SUR1 and SUR2A had no effect on the instantaneous MgATP concentration–response relationship in the presence of 100 μm MgADP (Fig. 4 *C*).

### Is the tail of SUR sufficient to account for differences in metabolic sensitivity?

**Figure 5:**
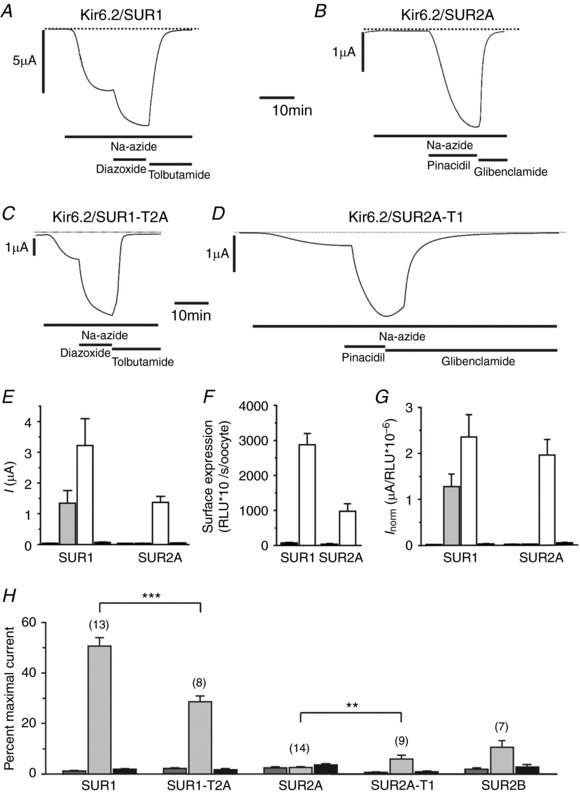
Metabolic inhibition of wild‐type and chimeric K_ATP_ channels *A*–*D*, representative whole‐cell currents recorded from *Xenopus* oocytes expressing Kir6.2/SUR1 (*A*), Kir6.2/SUR2A (*B*), Kir6.2/SUR1‐T2A (*C*) or Kir6.2/SUR2A‐T1 (*D*) channels. The horizontal bars indicate the presence of sodium azide (3 mm), K‐channel openers (340 μm diazoxide or 100 μm pinacidil) and sulphonylureas (0.5 mm tolbutamide or 50 μm glibenclamide). Different activators and inhibitors were used because pinacidil is specific for SUR2A and tolbutamide for SUR1. The dotted line indicates the zero current level. *E*, mean whole‐cell currents recorded before (dark grey bar) and after (grey bar) azide application, then after addition of a K‐channel opener (white bar) and finally in the presence of azide and a sulphonylurea (black bar). *F*, surface expression of Kir6.2‐HA/SUR1 and Kir6.2‐HA/SUR2A K_ATP_ channels (white bars) plotted as relative luminescence units (RLU) measured in the same batch of oocytes as in *E*. Black bars, control oocyte expressing Kir6.2/SUR1 and Kir6.2/SUR2A. *G*, mean whole‐cell currents normalised to the average RLU. *H*, mean whole‐cell currents recorded from *Xenopus* oocytes before (dark grey bar) and after (grey bar) azide application, and in the presence of azide + sulphonylurea (black bar), expressed as a percentage of that in the presence of azide + K‐channel opener. Numbers in parentheses denote the number of experiments. ^**^ *P *< 0.01; ^***^ *P *< 0.001.

To determine if differences in the tails of SUR1 and SUR2A account not only for the difference in nucleotide handling by the NBDs, but also for the metabolic sensitivity of the channel, we next examined the whole‐cell currents. As previously reported, Kir6.2/SUR1 but not Kir6.2/SUR2A channels were activated by metabolic poisoning with 3 mm azide (Fig. 5 *A*,*B*,*E*). This was not due to a failure of Kir6.2/SUR2A channels to express, because the K‐channel activator pinacidil produced a large current response. Surface expression assays also showed substantial expression (Fig. 5 *F*), and a similar relationship between protein expression and current amplitude in the presence of a channel activator for both types of channel (Fig. 5 *E–G*).

Kir6.2/SUR1‐T2A channels showed reduced metabolic activation compared to Kir6.2/SUR1 channels (*P* < 0.0001), although this remained substantially greater than that of Kir6.2/SUR2A (*P* < 0.0001) (Fig. 5 *C*,*H*): currents were expressed as a fraction of their amplitude in the presence of a K_ATP_ channel activator to account for any differences in expression. Kir6.2/SUR1‐T2A channels also activated with a similar time course to Kir6.2/SUR1 channels: τ = 153 ± 13 s (*n* = 8) *vs*. τ = 152 ± 9 s (*n* = 14), respectively (Fig. 5 *A vs*. Fig. 5 *C*). Kir6.2/SUR2A‐T1 channels gained some metabolic sensitivity but this was still substantially smaller than that of Kir6.2/SUR1 channels (*P* < 0.001; Fig. 5 *D*,*H*). In addition, the time course of activation of Kir6.2/SUR2A‐T1 channels was slower than that of Kir6.2/SUR1 and rarely reached a steady state. In these respects, Kir6.2/SUR2A‐T1 channels resemble Kir6.2/SUR2B channels: indeed, there was no significant difference between Kir6.2/SUR2A‐T1 and Kir6.2/SUR2B channels (Fig. 5 *H*).

### Complete deletion of the tail

**Figure 6:**
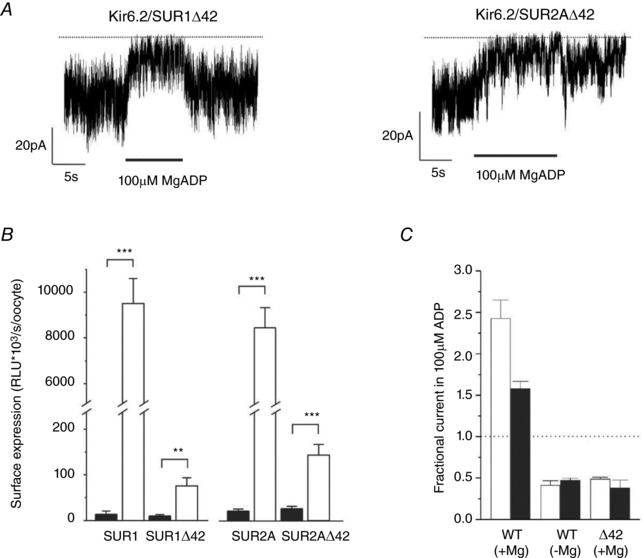
The last 42 residues of SUR are required for MgADP activation *A*, representative Kir6.2/SUR1Δ42 (left) and Kir6.2/SUR2AΔ42 (right) currents recorded at −60 mv in inside‐out patches from *Xenopus* oocytes; 100 μm MgADP was added as indicated. The dotted line indicates the zero current level. *B*, surface expression of HA‐tagged (white) and untagged (black) Kir6.2/SUR1, Kir6.2/SUR1Δ42, Kir6.2/SUR2A and Kir6.2/SUR2AΔ42 K_ATP_ channels, plotted as relative luminescence units (RLU) (*n* = 6–13). ^**^ *P* < 0.01; ^***^ *P* < 0.001. *C*, fractional currents remaining in the presence of 100 μm ADP recorded at −60 mV in inside‐out patches from *Xenopus* oocytes expressing wild‐type (WT) or truncated (Δ42) K_ATP_ channels, in the presence or absence of Mg^2+^. White bars: SUR1‐containing channels; black bars: SUR2A‐containing channels. Current is expressed as a fraction of that in nucleotide‐free solution (*n* = 6–13).

To gain further insight into the role of the SUR tail in the K_ATP_ channel function, we also examined the effect of deleting the last 42 amino acids of SUR1 or SUR2A (Fig. 6). For both channel types, this markedly decreased the macroscopic K_ATP_ current amplitude in excised patches (Fig. 6 *A*) and lowered the channel surface density ∼100‐fold (Fig. 6 *B*). This is consistent with studies showing that removing a C‐terminal dileucine forward trafficking motif in SUR1 impairs surface expression of the channel (Sharma *et al*. 1999). Tail deletion also reduced the instantaneous *P*_O_ in the absence of nucleotides for both types of channel (Table 2), which provides further support for the idea that the C‐terminal tail of SUR is important for the open state stability of the channel.

Surprisingly, tail deletion also completely abolished MgADP activation of both types of channel (Fig. 6 *A*,*C*). This result was unexpected because deleting the final 42 amino acids of SUR does not compromise the ability of the isolated NBDs to hydrolyse ATP (de Wet *et al*. 2010). This suggests that nucleotide binding/hydrolysis may no longer be coupled to channel activation when SUR is truncated, and thus that the final 42 amino acids may be necessary for transduction of nucleotide binding into channel activation.

## Discussion

Our data demonstrate a striking difference in nucleotide handling between Kir6.2/SUR2A and Kir6.2/SUR1 channels, and show that this involves the C‐terminal region of the channel.

### Reduced inhibitory effect of ATP in the presence of MgADP in excised patches

Although Kir6.2/SUR2A and Kir6.2/SUR1 channels are inhibited by ATP and activated by MgADP to similar extents and with similar *IC*_50_ and *EC*_50_ when these mechanisms are studied in isolation, when both nucleotides are simultaneously present channel regulation is strikingly different. This difference is particularly dramatic when the instantaneous ATP sensitivity is compared: in the presence of 100 μm MgADP, the *IC*_50_ for ATP inhibition of Kir6.2/SUR2A is almost 10‐fold less than that of Kir6.2/SUR1. As a consequence, the Kir6.2/SUR1 current at 1 mm MgATP is ∼30% of that in the absence of ATP whereas the Kir6.2/SUR2A current is < 0.2%. Except in conditions of very severe metabolic deficiency, ATP levels do not fall below 1 mm, and MgADP levels are < 100 μm. Thus our results may help to explain why β‐cell K_ATP_ channels open in the absence of glucose and cardiac K_ATP_ channels do not.

Our data demonstrate that there are intrinsic differences between SUR2A and SUR1 that account for the marked differences in the nucleotide and metabolic sensitivity when Kir6.2/SUR1 (β‐cell) and Kir6.2/SUR2A (cardiac) channels are expressed in *Xenopus* oocytes. It is also possible that additional regulatory mechanisms contribute to the differences in metabolic sensitivity observed in native cells: for example, the levels of phosphatidylinositol 4,5‐bisphosphate (PIP_2_) or the activity of the creatine phosphate/creatine kinase ATP buffering system (Li *et al*. 2002; Tarasov *et al*. 2006).

Previous studies have shown that in the presence of 3 mm sodium azide, ATP levels fall to ∼1.2−1.4 mm in *Xenopus* oocytes (Gribble *et al*. 2000; Li *et al*. 2016). This corresponds to a K_ATP_ current 20–23% of the maximum observed for Kir6.2/SUR1 in excised patches exposed to 100 μm MgADP (Fig. 3 *B*). It is in reasonable agreement with the *P*_O_ of Kir6.2/SUR1 channels, recorded from cell‐attached patches in cells exposed to 3 mm sodium azide, which is ∼30% of that of the maximally open channel (*P*_O_ of 0.86; Proks *et al*. 2010).

Our results indicate that the efficacy of MgADP bound to NBS2 of SUR1 to reduce ATP inhibition at Kir6.2 is substantially impaired by a combination of rundown and DAMN following patch excision. The extent of this effect can be estimated using a simple (Monod–Wyman–Changeux, MWC) model for concerted gating of K_ATP_ channels (Drain *et al*. 2004; Craig *et al*. 2008):

(3) $$P_{O}=\frac{1}{1+F+E}$$

(4) $$\begin{array}{ccc} & & \frac{P_{O}\left(IC_{50}\right)}{P_{O}}=\frac{1}{2}=\\ & & \frac{\left(1+F+E\right)*{\left(1+\frac{IC_{50}}{K_{d,O}}\right)}^{4}}{\left(1+F\right)*{\left(1+\frac{IC_{50}}{K_{d,O}}\right)}^{4}+E*{\left(1+\frac{IC_{50}}{K_{d,C}}\right)}^{4}} \end{array}$$

where *P*_O_ is the open probability in the absence of the nucleotide and *P*_O_(*IC*_50_) is open probability at the ATP concentration where inhibition is half maximal (*IC*_50_). *E* and *F* (0.15, from the maximal open channel probability) are the equilibrium gating constants for ‘slow’ and ‘fast’ gating of the channel (Proks & Ashcroft, 2009) and *K*_d,C_ and *K*_d,O_ (300 μm; Craig *et al*. 2008) are the dissociation constants for ATP binding to the closed and open states, respectively. MgADP binding to NBS2 stabilises the open state of the channel (Proks *et al*. 2010) and this effect can be modelled as

(5) $$E_{ADP}=\frac{E}{\varsigma }$$

where *E*_ADP_ is the equilibrium constant for ‘slow’ gating when MgADP is bound to SUR and ζ is a proportionality factor that accounts for the effect of MgADP on slow gating of the channel. Assuming that the stimulatory effect of MgADP on the channel is entirely due to gating, it is possible to calculate values for *E* and *K*_d,C_ from *P*_O_ and *IC*_50_ in the absence of MgADP (Tables 1 and 2) using eqns (3) and (4). The parameter ζ can then be determined from the value of *IC*_50_ in the presence of MgADP (Table 1). The model predicts ζ values of 1896 and 94 for the instantaneous and rundown experimental conditions, respectively. In other words, rundown/DAMN produces a ∼20‐fold reduction in the efficacy of MgADP.

An alternative possibility is that MgADP stimulation not only affects gating but also directly reduces ATP binding to Kir6.2 (Nichols *et al*. 1996; Shyng *et al*. 1997; Abraham *et al*. 2002). In this case, *K*_d,C_ and *K*_d,O_ could also be affected. It is not possible to distinguish formally between these two alternatives from the data.

The MWC model predicts that there is only a 4.6‐fold reduction in the parameter *E* when MgADP binds to SUR2A if the channel has rundown. Such a weak effect on gating might perhaps explain why MgADP has no effect on the *IC*_50_ for ATP inhibition of cardiac K_ATP_ channels measured immediately after patch excision (Fig 2 and Table 1), when the stimulatory effect of MgADP at SUR2A on gating may be compromised by the inhibitory effect of ADP at Kir6.2. Note that MgADP causes a small decrease (from 0.86 to 0.84) in the *P*_O_ of Kir6.2/SUR2A channels immediately after patch excision (Table 2).

### Effects of tail deletion

Deletion of the last 42 residues of either SUR1 or SUR2A impaired surface expression of the K_ATP_ channel and reduced its intrinsic open probability. It also prevented MgADP activation. Instead, MgADP inhibited both types of channel. This inhibition represents inhibition at Kir6.2, as the extent of inhibition was the same as that seen for wild‐type channels in the absence of Mg^2+^. It has been previously reported that deletion of the C‐terminus does not impair MgATP hydrolysis by purified NBD2 (de Wet *et al*. 2010), indicating MgATP binding is unaffected in the isolated NBD. With the caveat that the NBDs may behave differently in the channel complex than in isolation, our results suggest that deletion of the tail of either SUR1 or SUR2A impairs the ability of bound MgADP to exert its stimulatory effect on channel opening. This idea is supported by the cryo‐electronmicroscopy structure of the K_ATP_ channel with Mg‐nucleotides bound to the NBSs of SUR1 (Lee *et al*. 2017). In this structure, the C‐terminal region of SUR1 lies close to the Walker A motif of NBS2, which is itself in very close proximity to Kir6.2 (20−30 nm; Lee *et al*. 2017). Thus, the C‐terminus of SUR may be involved in coupling occupancy of the NBDs to gating of the Kir6.2 pore. For example, it may influence the ability of the NBDs to dimerise and thereby induce the conformational changes that couple MgADP binding to channel opening. This would explain why deletion of the tail prevents MgADP activation. If the tail of SUR2A was less effective at promoting dimerisation, it could also help explain the difference in the efficacy of MgADP at SUR1 and SUR2A in promoting channel activity.

### The C‐terminus contributes to functional differences in SUR1 and SUR2A

Our results demonstrate that the difference in the ability of MgADP to reduce the ATP sensitivity of Kir6.2/SUR1 and Kir6.2/SUR2A channels resides in the last 42 amino acids of SUR. This is because replacing the tail of SUR1 with that of SUR2A resulted in channels that behaved like SUR2A, and vice versa. It may also explain why Kir6.2/SUR2B, in contrast to Kir6.2/SUR2A, shows a dramatic reduction in ATP inhibition in the presence of 100 μm MgADP (Table 1) – the tail of SUR2B is ∼75% homologous to that of SUR1 but only 30% homologous with that of SUR2A.

Although exchanging the C‐terminal tails of Kir6.2/SUR1 and Kir6.2/SUR2A channels swapped the instantaneous ATP sensitivity in the presence of MgADP, it only partially recapitulated the increase in K_ATP_ current produced by sodium azide. Thus, stimulation of K_ATP_ channel activity upon metabolic poisoning is likely to also involve additional regions of the channel, additional mechanisms or auxiliary proteins.

## Conclusions

We conclude that different efficacies of MgADP bound at SUR1 and SUR2A to reduce ATP inhibition at Kir6.2 partially explain the different metabolic sensitivities of Kir6.2/SUR1 and Kir6.2/SUR2A channels. The underlying mechanism for this effect involves the C‐terminal 42 amino acids of SUR. Deletion of these residues abolishes MgADP activation. This suggests that the C‐terminus of SUR may be involved in stabilising MgADP binding, preventing closure of the NBD dimer, or decreasing the coupling efficacy between MgADP occupancy, closure of NBD dimer and gating of the Kir6.2 pore (or potentially all of these possibilities). Nevertheless, when taken together with previous studies, our results favour the view that the C‐terminus of SUR is involved in transducing occupancy of the NBDs of SUR to gating of the Kir6.2 pore.

## Additional information

### Competing interests

The authors declare no competing financial interests.

### Author contributions

Conceptualisation: FMA, PP and NV; data acquisition and analysis: PP, KH, OHR and NV; writing: FMA, PP and NV. All authors have read and approved the final version of the manuscript.
